# Draft Genome Sequence of the Oleaginous Yeast *Saitozyma podzolica* (syn. *Cryptococcus podzolicus*) DSM 27192

**DOI:** 10.1128/MRA.01676-18

**Published:** 2019-02-21

**Authors:** Habibu Aliyu, Olga Gorte, Anke Neumann, Katrin Ochsenreither

**Affiliations:** a Institute of Process Engineering in Life Science 2: Technical Biology, Karlsruhe Institute of Technology, Karlsruhe, Germany; University of California, Riverside

## Abstract

We report here the draft genome of Saitozyma podzolica DSM 27192 sequenced based on PacBio chemistry. This yeast isolate produces large amounts of single-cell oil (SCO) and gluconic acid (GA). Information from the genome sequence will provide additional insight into the genetic mechanism of SCO and GA metabolism in this organism.

## Abstract (precis)

We report here the draft genome of Saitozyma podzolica DSM 27192 sequenced based on PacBio chemistry. This yeast isolate produces large amounts of single-cell oil (SCO) and gluconic acid (GA).

## ANNOUNCEMENT

A yeast isolate from peat bog soil in the Black Forest of Germany was identified using internal transcribed spacer (ITS) region sequencing as Cryptococcus podzolicus and deposited as DSM 27192 at the DSMZ (1). This oleaginous yeast was characterized as a simultaneous single-cell oil (SCO) and gluconic acid (GA) producer via a fermentation process with glucose as a carbon source, yielding 18 g/liter SCO and 30 g/liter GA. On xylose, 11.1 g/liter SCO was produced (1). The ability to convert xylose to SCO makes this strain an interesting candidate for the maintenance of the carbon value chain by converting renewable waste material, e.g., hydrolyzed wood and straw, for worthwhile biodiesel production (2).

Saitozyma podzolica DSM 27192 was cultivated in a mineral salt medium described in reference 1. Genomic DNA was extracted from cells grown to early logarithmic phase at 20°C and 130 rpm. Genomic DNA was isolated using a phenol-chloroform protocol (3). Library preparation and PacBio RS long-read sequencing were performed at GATC Biotech AG (Constance, Germany), yielding 203,613 reads of 2,572,991,431 bp, with an *N*_50_ value and mean read length of 19,167 and 12,636 bp, respectively. The reads were preassembled using HGAP 3.0 and polished using Quiver as part of SMRT Analysis version 2.3 at GATC Biotech AG. To validate this assembly, the reads were assembled and polished using Canu version 1.7.1 (4) and arrow version 2.3.2 (5), respectively. The final assembly comprises 46 contigs of 29,888,215 bp (86.1× coverage) with a mean G+C content of 58.79%. The sizes of the largest and *N*_50_ contigs were 2,705,151 and 1,066,819 bp, respectively. In comparison, the DSM 27192 draft genome is larger than the genomes of other members of the genera Dioszegia, Saitozyma, and Tremella, whose genome sizes range from 19,344,119 to 28,639,919 bp.

**FIG 1:**
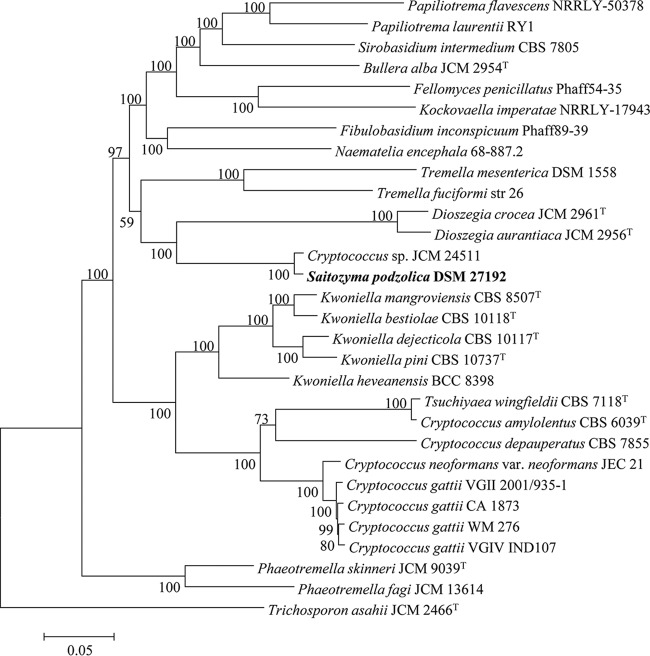
Core genome phylogeny of 31 representative members of the order Tremellales and Trichosporon asahii JCM2466^T^. Maximum likelihood (ML) tree inferred from concatenated protein alignment (45,703 amino acids in length) of 164 single-copy proteins. The phylogeny was generated using IQ-TREE version 1.6.7 based on the LG+F+R5 model (8). The ML was generated with confidence values based on 1,000 bootstrap replicates.

Annotation of DSM 27192 contigs using Funannotate pipeline version 1.5.0-8f86f8c (6) resulted in 10,312 gene predictions comprising 10,224 protein-coding and 88 tRNA genes. Compared to its closest genomic relative (Fig. 1), Cryptococcus sp. strain JCM 24511 (GenBank accession number BCLC00000000), the genome of DSM 27192 encodes 1,689 unique proteins. The completeness of the DSM 27192 draft genome, based on BUSCO (7) fungi_odb9 and basidiomycota_odb9, stands at 92.4% and 90.0%, respectively. In contrast, the completeness of the Cryptococcus sp. JCM 24511 genome, based on fungi_odb9, was estimated at 94.8%. However, fungi_odb9 estimate of genome completeness among other members of the genera Dioszegia, Saitozyma, and Tremella ranged from 87.6 to 94.8%. Functional annotation of the predicted proteins revealed that relative to other members of the order Tremellales, DSM 27192 genome encodes a larger number of proteins linked to carbohydrate-active enzymes (CAZymes) and MEROPS, as well as secreted proteins.

The whole-genome sequence of S. podzolica DSM 27192 will provide additional insight into the genetic mechanisms and control of fatty acids and gluconic acid synthesis. At the same time, the genomic data will provide an additional point of comparison to other oleaginous yeasts and GA-producing organisms.

### Data availability.

This whole-genome shotgun project has been deposited at DDBJ/ENA/GenBank under the accession number RSCD00000000. The raw reads were deposited in the Sequence Read Archive under the accession number PRJNA506379.

## References

[1] Schulze I, Hansen S, Großhans S, Rudszuck T, Ochsenreither K, Syldatk C, Neumann A 2014 Characterization of newly isolated oleaginous yeasts–Cryptococcus podzolicus, Trichosporon porosum and Pichia segobiensis. AMB Express 4:24. doi:10.1186/s13568-014-0024-0. 24949259 PMC4052688

[2] Li Q, Du W, Liu D 2008 Perspectives of microbial oils for biodiesel production. Appl Microbiol Biotechnol 80:749–756. doi:10.1007/s00253-008-1625-9. 18690426

[3] Sambrook J, Russell DW 2006 Purification of nucleic acids by extraction with phenol: chloroform. CSH Protoc 2006:pdb.prot4455. 22485786 10.1101/pdb.prot4455

[4] Koren S, Walenz BP, Berlin K, Miller JR, Bergman NH, Phillippy AM 2017 Canu: scalable and accurate long-read assembly via adaptive k-mer weighting and repeat separation. Genome Res 27:722–736. doi:10.1101/gr.215087.116. 28298431 PMC5411767

[5] Chin C-S, Alexander DH, Marks P, Klammer AA, Drake J, Heiner C, Clum A, Copeland A, Huddleston J, Eichler EE, Turner SW, Korlach J 2013 Nonhybrid, finished microbial genome assemblies from long-read SMRT sequencing data. Nat Methods 10:563. doi:10.1038/nmeth.2474. 23644548

[6] Palmer J 2016 Funannotate: pipeline for genome annotation. https://zenodo.org/record/1471785#.XFO5kvlKi1t.

[7] Waterhouse RM, Seppey M, Simão FA, Manni M, Ioannidis P, Klioutchnikov G, Kriventseva EV, Zdobnov EM 2018 BUSCO applications from quality assessments to gene prediction and phylogenomics. Mol Biol Evol 35:543–548. doi:10.1093/molbev/msx319. PMC5850278 29220515

[8] Chernomor O, von Haeseler A, Minh BQ 2016 Terrace aware data structure for phylogenomic inference from supermatrices. Syst Biol 65:997–1008. doi:10.1093/sysbio/syw037. 27121966 PMC5066062

